# Reassessment of the Enteropathogenicity of Mesophilic *Aeromonas* Species

**DOI:** 10.3389/fmicb.2016.01395

**Published:** 2016-09-21

**Authors:** Peter Teunis, Maria J. Figueras

**Affiliations:** ^1^Centre for Zoonoses and Environmental Microbiology, Centre for Infectious Disease Control, National Institute for Public Health and the Environment, BilthovenNetherlands; ^2^Center for Global Safe WASH, Hubert Department of Global Health, Rollins School of Public Health, Emory University, Atlanta, GAUSA; ^3^Unitat de Microbiologia, Departament de Ciènces Médiques Bàsiques, Facultat de Medicina i Ciències de la Salut, Pere Virgili Institute for Health Research, Universitat Rovira i Virgili, ReusSpain

**Keywords:** *Aeromonas*, outbreaks, water, food, challenge study, infective dose, risk of infection, risk of illness

## Abstract

Cases of *Aeromonas* diarrhea have been described all over the world. The genus *Aeromonas* includes ca. 30 species, of which 10 have been isolated in association with gastroenteritis. The dominating species that account for ca. 96% of the identified strains are *Aeromonas caviae, A. veronii*, *A. dhakensis*, and *A. hydrophila*. However, the role of *Aeromonas* as a true enteropathogen has been questioned on the basis of the lack of outbreaks, the non-fulfillment of Koch’s postulates and the low numbers of acute illnesses in the only existing human challenge study. In the present study we reassess the enteropathogenicity of *Aeromonas* using dose response models for microbial infection and acute illness. The analysis uses the data from the human challenge study and additional data from selected outbreak investigations where the numbers exposed and the dose were reported, allowing their inclusion as “natural experiments”. In the challenge study several cases of asymptomatic shedding were found (26.3%, 15/57), however, only 3.5% (2/57) of those challenged with *Aeromonas* developed acute enteric symptoms (i.e., diarrhea). The “natural experiments” showed a much higher risk of illness associated with exposure to *Aeromonas*, even at moderate to low doses. The median dose required for 1% illness risk, was ~1.4 × 10^4^ times higher in the challenge study (1.24 × 10^4^ cfu) compared to natural exposure events (0.9 cfu). The dose response assessment presented in this study shows that the combined challenge and outbreak data are consistent with high infectivity of *Aeromonas*, and a wide range of susceptibility to acute enteric illness. To illustrate the outcomes, we simulate the risk associated with concentrations of *Aeromonas* found in different water and food matrices, indicating the disease burden potentially associated with these bacteria. In conclusion this study showed that *Aeromonas* is highly infectious, and that human susceptibility to illness may be high, similar to undisputed enteropathogens like *Campylobacter* or *Salmonella*.

## Introduction

The genus *Aeromonas*, includes Gram-negative, non-spore-forming rods that are autochthonous of the aquatic environments worldwide (Austin et al., 1996; Janda and Abbott, 2010; Figueras and Beaz-Hidalgo, 2015; Graf, 2015). In humans diarrhea, and wound infections are the most common presentation followed by bacteremia (Figueras, 2005; von Graevenitz, 2007; Janda and Abbott, 2010; Figueras and Beaz-Hidalgo, 2015). Isolation of *Aeromonas* in patients with diarrhea varies from ca 2 to 10% but a higher incidence (13%) has been found in children from Nigeria (Figueras and Beaz-Hidalgo, 2015). Also a significantly higher incidence of *Aeromonas* is found in patients with diarrhea than in those considered asymptomatic carriers (Figueras, 2005; Janda and Abbott, 2010; Figueras and Beaz-Hidalgo, 2015). Typical susceptible patients in the different studies include babies, young children and the elderly and especially those with a pre-existing illness and/or immunocompromised (Janda and Abbott, 2010; Figueras and Beaz-Hidalgo, 2015). According to Janda and Abbott (2010) it is important to report the presence of *Aeromonas* in stools of immunocompromised patients even if they are only asymptomatic because the risk of invasion and dissemination is considered inherently high for these patients. Nevertheless the role of *Aeromonas* in gastroenteritis has been questioned (Janda and Abbott, 1998, 2010; Chu et al., 2006; von Graevenitz, 2007), mainly because 55 out 57 challenged volunteers with a high dose of *Aeromonas* did not developed any symptoms of enteric illness, and due to the few reported outbreaks (Morgan et al., 1985; Figueras et al., 2007b; Janda and Abbott, 2010; Figueras and Beaz-Hidalgo, 2015). However, there are also many publications providing support for a causal relation between *Aeromonas* and enteric disease (Figueras et al., 2007a,b; Janda and Abbott, 2010; Figueras and Beaz-Hidalgo, 2015). Although 10 of the ca. 30 species that comprise the genus have been isolated in association with gastroenteritis, only 4, i.e., *Aeromonas caviae, A.* *veronii*, *A. dhakensis*, and *A.* *hydrophila* are the dominating species accounting for ca. 96% of the recovered isolates from this origin in different studies (Figueras and Beaz-Hidalgo, 2015).

Consumption of contaminated water and food are considered the main routes of transmission and there are few reports of well documented outbreaks that include information about the ingested doses of *Aeromonas* (Krovacek et al., 1995; Granum et al., 1998; Zhang et al., 2012). An association between diarrheal cases and consumption of untreated or contaminated drinking water has been established in several occasions (Holmberg et al., 1986; Martin Delgado et al., 2001; Khajanchi et al., 2010; Pablos et al., 2011; Ventura et al., 2015). *Aeromonas* was isolated from drinking water and stools in some patients that were diagnosed with traveler’s diarrhea among the tourists that visited specific hotels in the coastal area of Tenerife and Canary Islands in Spain (Martin Delgado et al., 2001). Failures in the drinking water distribution system caused fecal contamination of the water, as revealed by the presence of bacterial indicators of fecal pollution, high concentrations of organic matter and the detection of *Aeromonas* (Martin Delgado et al., 2001). Once the water distribution system was repaired the incidence of diarrhea among the tourist population returned to normal. Even a recurrent case of *Aeromonas* bacteremia has been attributed to the consumption of contaminated well water (Katz et al., 2015). Use of contaminated water can cause secondary contamination of food products, and this can be the source of food-borne outbreaks (Altwegg et al., 1991; Krovacek et al., 1995; Granum et al., 1998; Figueras and Borrego, 2010; Wadhwa et al., 2012; Zhang et al., 2012; Figueras and Beaz-Hidalgo, 2014).

It has become generally accepted that only a subset of *Aeromonas* strains can cause gastroenteritis in humans (von Graevenitz, 2007; Janda and Abbott, 2010; Grim et al., 2014). However, nowadays it is clear that infection is a complex process in which not only the virulence of the colonizing strain is important, but also its interaction with other microbes that are present in the gut, as co-infecting pathogens or in the natural microbial ecosystem, together with the specific physiological status of the host (Lund and O’Brien, 2011; Leggett et al., 2012; Beaz-Hidalgo and Figueras, 2013; Mosser et al., 2015; Ribet and Cossart, 2015; Denny et al., 2016). In fact, the overall crosstalk and interactions between commensal bacteria, enteric pathogens, and host physiology is what is considered crucial to the establishment and progression of intestinal disease (Leggett et al., 2012; Ribet and Cossart, 2015).

Challenge studies have been essential, to establish a causal relation between exposure and health effects and for quantifying the dose response relation (Teunis et al., 1996; Teunis and Havelaar, 2000). However, some outbreak reports include data on the numbers of person exposed, and even information allowing an estimate of the magnitude of the dose involved. Such outbreaks may be treated as ’natural experiments or natural exposure’, comparable to a challenge experiment with a single dose group (Teunis et al., 2004, 2008, 2012b). Both data sources have been successfully used in several studies that investigated the potential of human pathogens different from *Aeromonas* for producing colonization (infectivity) and acute illness (pathogenicity) (Teunis et al., 2005, 2010).

In the present study we use dose response models for microbial infection and acute illness (Teunis et al., 1999; Teunis and Havelaar, 2000) to re-assess the Morgan et al. (1985) challenge study and combine these data with outbreak investigations to determine the dose response relation for producing infection and acute enteric disease by *Aeromonas* spp. Furthermore, to illustrate the outcomes, we estimate the risk associated with waterborne exposure to these bacteria using reference concentrations found in different water or food matrices. In addition we provide evidence in support of *Aeromonas* acting as a true enteropathogen.

## Materials and Methods

### Data Used in the Dose Response Model

The volunteer protocols for the Morgan et al. (1985) human challenge study were approved by the institutional review boards of the University of Texas Health Science Center, Baylor College of Medicine, The Methodist Hospital and the General Clinical Research Center, and as common in a dose response study, did not include a non-exposed control group. The five different *Aeromonas* strains that were administered orally in a bicarbonate solution to 57 adult healthy human volunteers at a range of doses and the outcomes obtained (Morgan et al., 1985) are summarized in **Table 1**. A prechallenge stool was cultured for enteropathogens from all volunteers before admission, and volunteers abstained from eating and drinking 90 min before and after oral challenge. Volunteers were followed up with a daily physical examination to determine symptoms of gastroenteritis (defined as 2 or more unformed stools in 24 h accompanied by any symptoms of enteric disease). Infection was defined as fecal shedding of the inoculated strain detected by analysis of the volunteer’s feces.

**Table 1:** Strains of *Aeromonas* and doses challenged to different groups of volunteers (data from Morgan et al., 1985).

	Dose	Volunteers		
Strain	(cfu)	Challenged	Infected	Illness
6Y	2 × 10^4^	4	1	0
	1 × 10^6^	4	1	0
	7 × 10^7^	4	4	0
	3 × 10^9^	4	3	1
	4 × 10^10^	4	2	0
B158	6 × 10^4^	4	0	0
	2 × 10^7^	4	0	0
3647	1 × 10^7^	4	1^a^	1
	4 × 10^7^	4	0	0
	2 × 10^9^	4	2	0
	3 × 10^10^	4	1	0
SSU	4 × 10^8^	4	0	0
	5 × 10^10^	3	0	0
3284	3 × 10^8^	3	0	0
	1 × 10^10^	3	0	0

*^a^According to Morgan et al. (1985), there was not infection but it was only acute illness, however, this could be either a false negative or a false positive (as explained in the materials and methods). Given the setting of inoculation with a high dose, we consider the first alternative (false negative infection) most plausible.*

One of the subjects who was challenged with strain 3647 developed acute enteric symptoms, while no shedding of the inoculated strain could be detected. This outcome could indicate a false positive for symptoms, if the enteric symptoms in this subjects were caused by something different, unrelated to the challenge. It could also be a false negative infection, where this subject had been colonized without detectable pathogen shedding. Such false negative shedding has been reported in other challenge studies (DuPont et al., 1969), possibly indicating intermittent shedding or low sensitivity of the detection method. Given the setting of inoculation with a high dose, we consider the second alternative (false negative infection) most plausible. In addition to these clinical experiment data, a literature review was done to collect data on acute enteric illness caused by *Aeromonas* due to natural exposures or outbreaks (here called “natural experiments”). Only studies where sufficient information was reported to allow a dose response assessment were selected. Four “natural experiments” with these characteristics were found: 3 foodborne outbreaks (Krovacek et al., 1995; Granum et al., 1998; Zhang et al., 2012) and an incident where a laboratory worker had accidentally ingested a pure culture of 10^9^ *Aeromonas* (Carnahan et al., 1991). The data used from those studies are summarized in **Table 2**. To our knowledge these are the only reports that include numbers of exposed subjects and numbers of acute cases, and information on exposure, either as estimated numbers of ingested bacteria or as amount of the contaminated food product consumed (**Table 2**). The foodborne outbreak reported by Zhang et al. (2012) showed a dose response relation between the amounts of food consumed (cucumber salad) and the observed attack rate.

**Table 2:** Dose and/or concentrations of *Aeromonas* found in the ingested matrices and number of exposed and cases showing acute gastrointestinal symptoms in outbreaks or natural experiments.

Reference	Matrix	Intake	Concentration cfu/g	Exposed	Ill
Zhang et al., 2012^a^	Cucumber salad	2 tbsp	NA	178	73
		1 tbsp	NA	153	59
		0.5 tbsp	NA	141	39
Krovacek et al., 1995^b^	Swedish salad	50 g	10^6^–10^7^	27	24
Granum et al., 1998^b^	Fermented fish	10^7^	4	3
Carnahan et al., 1991^b^	Broth of a pure culture	10^9^

**FIGURE 1:**
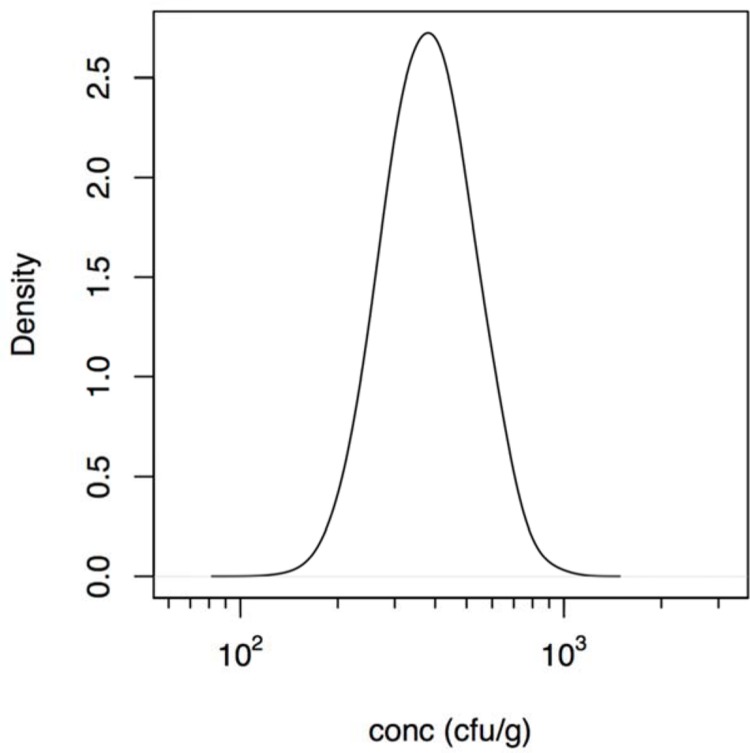
**Estimated (posterior) distribution of the concentration (cfu/g) of *Aeromonas* in the cucumber salad derived using the intake data described in the outbreak reported by Zhang et al. (2012)**.

*NA = not available; tbsp = table spoon; ^a^Intake of food (cucumber salad) with unknown concentration, so the concentration was estimated in this study (**Figure 1**). ^b^The concentration is the dose given by the authors.*

### Dose Response Assessment

Conceptually, three stages may be identified when a subject is challenged: exposure, infection, and (acute) illness. For exposure, it is important to realize that many microbial pathogens are highly infectious. Whenever a person ingests a quantity of contaminated food or drink, they only need to swallow few pathogenic particles to become infected. If the concentration of pathogens in the contaminated food or drink is low, the probability that the ingested portion did not contain any pathogens may be substantial. If (and only if) a person is exposed, there is a non-zero probability that any of the ingested pathogens survives all host barriers and succeeds in colonizing host tissues (i.e., infection is conditional on exposure). Likewise, if (and only if) a person is infected, there is a non-zero probability that the colonizing pathogen expresses pathogenicity producing damage of host tissues leading to symptoms of (acute) illness (i.e., illness is conditional on infection). Thus, there are two conditional probabilities: the probability of infection given exposure, and the probability of (acute) illness given infection. Both probabilities may be dose dependent, so that there are two dose response relations: one for infection, and the second for illness among infected subjects. Mathematical details for these two dose response relations are given in the Supplementary Material, where it is also indicated that all analyses were performed using JAGS (v4.2.0), with post-processing (graphs and additional statistics) in R (v3.3.1).

The parameters for these dose response relations, characterizing susceptibility to infection and to illness when infected, were estimated in a hierarchical framework. Each strain in the challenge study was treated as a separate trial, leading to strain-specific dose response relations for infection and illness. Similarly, each “natural experiment” was treated separately, as described by its own distinct dose response relations, for infection and illness. In the hierarchical framework, the variation in the parameters among these separate dose response relations is described by a (joint) distribution, representing the “group” pattern for all studies combined (Teunis et al., 2008). Hence, using all studies combined, it is possible to make predictions of the dose response relations for infection and illness, for any *Aeromonas* as representing a random sample from a population characterized by the combined studies shown in **Tables 1** and **2**.

In a subsequent analysis the challenge studies and the natural experiments were compared by assigning each of these two categories different susceptibilities to infection and illness. These separate predictions of the dose response relations for the challenge study strains and for the natural exposure events may thus be compared.

Note that in the foodborne outbreak described by Zhang et al. (2012) the intake of contaminated food was reported, but not the concentration of bacteria or its possible inhomogeneous distribution in that food due to clustering occurrence. Assuming the dose is proportional to the amount consumed, the concentration of bacteria may be added as another parameter (Teunis et al., 2005). This allows estimation of the mean dose (show in **Figure 1**) even in case there is heterogeneity because of an uneven distribution of bacteria (clustering) in the contaminated food. Unfortunately, the information available does not allow quantitative characterization of this heterogeneity as done in previous studies (Teunis et al., 2008).

### Estimation of Risk

To illustrate the use of the inferred dose response models, Monte Carlo samples of the dose response parameters for infection and (conditional) illness were used to calculate risks of infection and (acute enteric) illness for a few exposure scenarios. The probabilities of infection and illness resulting from exposure to low, medium, and high doses (10, 1000, and 10^6^ cfu) of *Aeromonas* were calculated, using the dose response models for infection and illness (see annex Supplementary Material). The selected scenarios represent concentrations of *Aeromonas* commonly found in drinking water distribution systems or food products like milk, meat products or shellfish (Abeyta et al., 1986; Austin et al., 1996; Borrell et al., 1998; Figueras et al., 2005; Egorov et al., 2011; Wadhwa et al., 2012; Robertson et al., 2014) or contaminated water (rivers, lakes) with treated or untreated wastewater (Austin et al., 1996; Borrell et al., 1998; McMahon and Wilson, 2001; Janda and Abbott, 2010; Latif-Eugenín, 2015; Fernandez-Cassi et al., 2016).

## Results

### Dose Response Assessment

The calculated estimated concentration of *Aeromonas* in the cucumber salad that was the vehicle for exposure in the outbreak reported by Zhang et al. (2012), is shown in **Figure 1** and ranged between 200 and 1000 cfu/g. So the likely dose in that outbreak was lower than the doses in the challenge study (see Supplementary Material for details).

**FIGURE 2:**
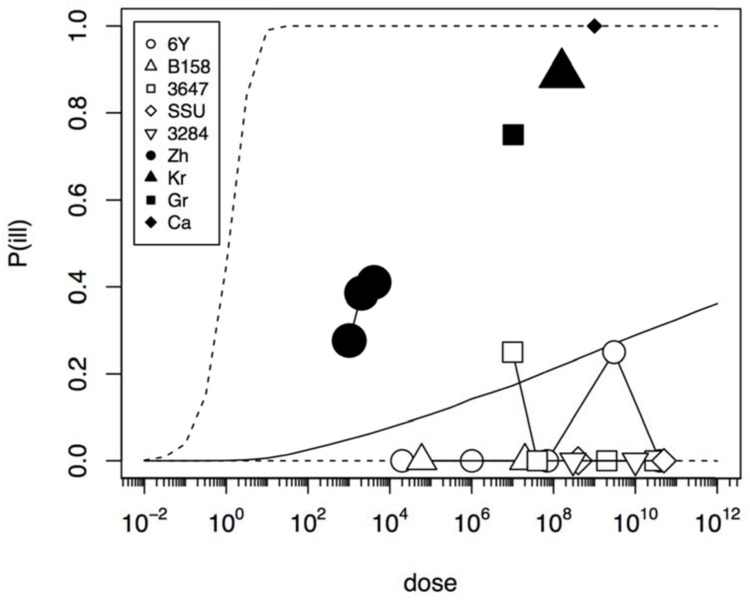
**Predicted dose response relation for acute enteric illness assuming a single class of infectivity and pathogenicity.** Median probability of illness (solid black line) and 95% predictive intervals (dotted lines). The five strains (6Y, B158, 3647, SSU, and 3284) used in the challenge study show a low virulence (data in **Table 1**), and the four natural experiments (Zh = Zhang et al., Kr = Krovacek et al., Gr = Granum et al., Ca = Carnahan et al.) show a high virulence (data in **Table 2**). Plot symbols show observed fractions, data from the same strain/experiment are connected. Sizes of symbols indicate numbers of subjects exposed.

**FIGURE 3:**
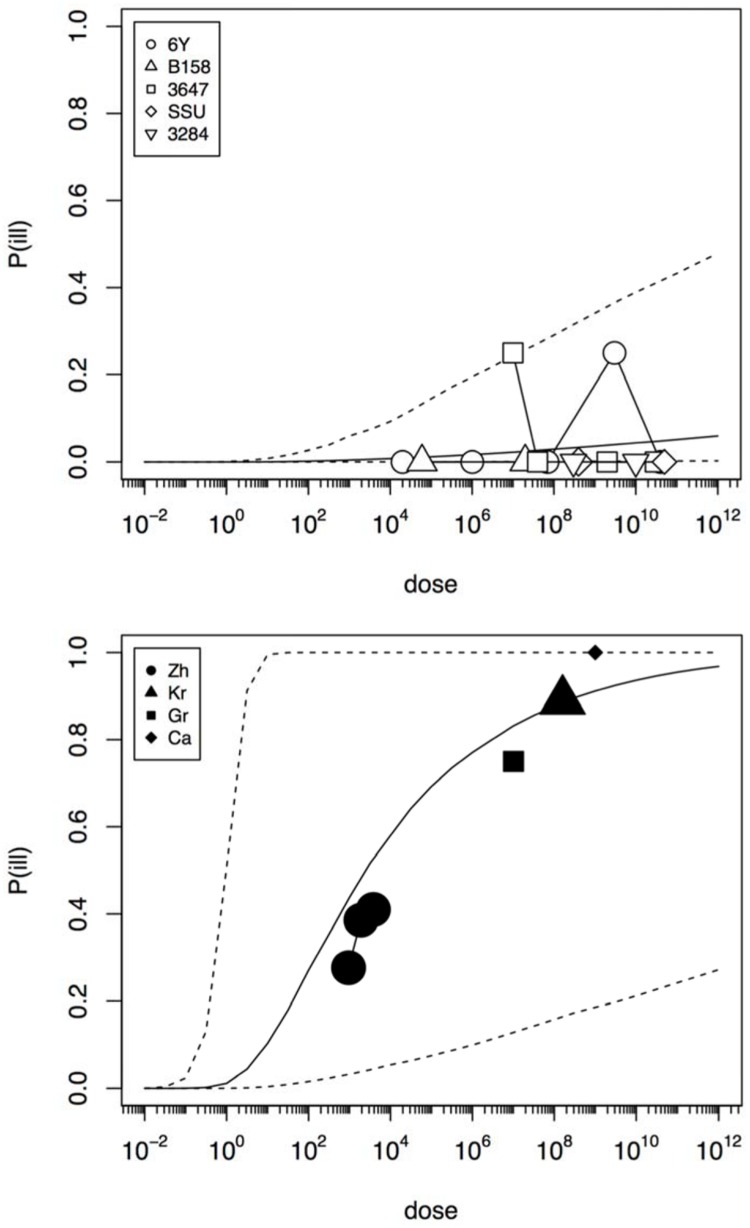
**Predicted dose response relations for acute enteric illness, derived from the five strain (6Y, B158, 3647, SSU, and 3284) of the challenge study (upper graphic showing a low susceptibility) and the four natural experiments (Zh = Zhang et al., Kr = Krovacek et al., Gr = Granum et al., Ca = Carnahan et al., lower graphic showing a high susceptibility).** Median probability of illness (solid black line) and 95% predictive intervals (dotted lines). Plot symbols show observed fractions, data from the same strain/experiment are connected. Sizes of symbols indicate numbers of subjects exposed.

In an initial analysis the data from the challenge study and the outbreaks (natural experiments or natural exposure events) were assumed to be similar and were represented by single joint distributions (**Figure 2**) that showed the susceptibility to infection (i.e., the probability of infection per ingested bacteria) and to illness (i.e., the probability of illness per ingested bacteria). This unsegmented approach resulted in a single predicted dose-response relation for illness, as show in **Figure 2**, with a broad posterior range in illness probabilities, indicating substantial heterogeneity in illness risk. In **Figure 2** it can be observed that the data from the “natural experiments” (i.e., outbreaks or natural exposure events) cluster at higher illness probabilities compared to the challenge study data. Based on these different outcomes, a refined model was set up, with separate categories for the challenge study and the “natural experiments” that represented two different distributions of susceptibility. The resulting separate dose response models for the challenge study and “natural exposure events” were markedly different, as shown in **Figure 3**. For the challenge study, ingestion of even very high doses of 10^12^ cfu results in low risk of acute illness (approximately 0.1, 95% range 0–0.45) as shown in **Figure 3**. Note, however, that even this low risk is caused by only two positive responses, with two different strains (3647 and 6Y), out of a total of 57 subjects exposed. In an alternative analysis where the symptomatic case for strain 3647 was considered a false positive, the resulting dose response models (not shown here) were virtually identical to those in **Figures 2** and **3**. Of those exposed, 15 were infected, indicating that infection was a lot more common than symptomatic acute illness, and also that pathogenicity may have been different among strains. For the “natural exposure events” the illness risk was much higher (approximately 0.5, 95% range 0.05–1.0), even at a comparatively low dose of 10^3^ cfu (**Figure 3**, lower graphic).

**FIGURE 4:**
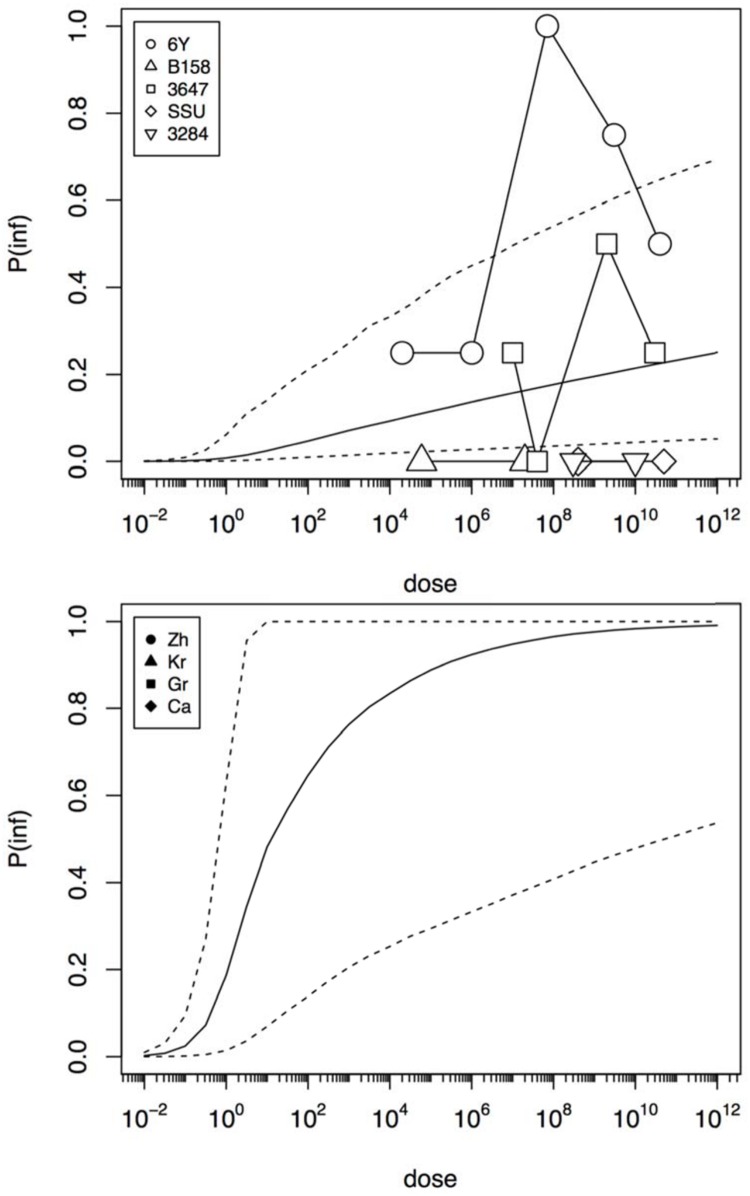
**Predicted dose response relations for infection, derived from the five strain (6Y, B158, 3647, SSU, and 3284) of the challenge study (upper graphic showing a low susceptibility) and the four natural experiments (Zh = Zhang et al., Kr = Krovacek et al., Gr = Granum et al., Ca = Carnahan et al., lower graphic showing a high susceptibility).** Median probability of infection (solid black line) and 95% predictive intervals (dotted lines). Plot symbols show observed fractions, data from the same strain/experiment are connected. Sizes of symbols indicate numbers of subjects exposed.

**Figure 4** shows infection dose response relations for the challenge study and the “natural exposure” data. As infection is a condition for illness, the probability of infection must be at least as high as the illness probability (compare **Figures 3** and **4**). The natural exposure event data do not include any observation of infections, as this is a covert outcome (it cannot be observed directly), but because illness depends on infection the probability of infection can still be estimated. As the illness risk was high in these “natural experiments”, the infection probabilities must be high as well, as shown in **Figure 4**.

**FIGURE 5:**
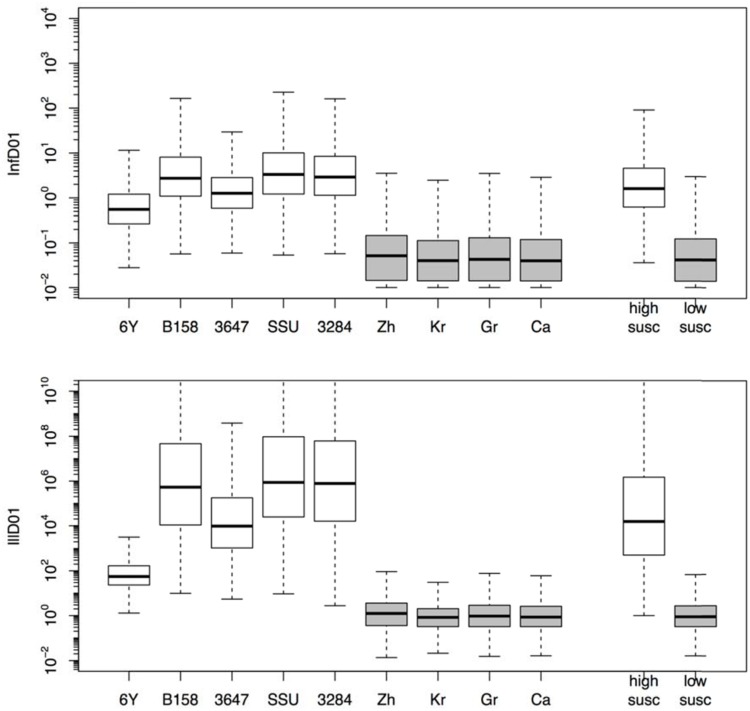
**Box plots showing the estimated doses required for 1% probability of infection (InfD01, upper graphic) and illness (IllD01, lower graphic).** Results in both graphics are provided for each individual strain (6Y, B158, 3647, SSU, and 3284) and experiment (Zh = Zhang et al., Kr = Krovacek et al., Gr = Granum et al., Ca = Carnahan et al.) and globally for all strains (i.e., low susceptibility) and natural experiments (i.e., high susceptibility).

The estimated susceptibilities for infection and illness were illustrated also by the doses required to cause a 1% probability of infection for each of the challenge strains, and separately for each of the “natural exposure events” (**Table 3**; **Figure 5**). Estimates for the two prediction categories: low susceptibility for the challenge studies and high susceptibility for the “natural experiments” are shown in **Figure 5** for infection and for illness risks. An outcome of 1% risk was chosen here because in the challenge studies the risk was so low that calculation of 50% infectious (and illness) doses results in unrealistically high numbers. Mean and median, as well as a 95% range are also given in **Table 3**.

**Table 3:** Doses (mean, median, and 95% CI) for 1% risk of infection and illness calculated for each individual strain/natural experiment, and globally for all strain/natural experiments (corresponding to the low/high susceptibility situations).

	1% Infectious dose			1% Illness dose		
	Mean	Median	95% CI	Mean	Median	95% CI
6Y^a^	1.04	0.56	0.07–4.95	1.47 × 10^4^	60.8	4.19–5.09 × 10^3^
B158^a^	1893.6	2.58	0.15–126.5	1.90 × 10^9^	4.97 × 10^5^	126.0–2.67 × 10^10^
3647^a^	2.69	1.27	0.12–14.6	2.94 × 10^8^	8400.7	53.6–1.54 × 10^9^
SSU^a^	412.1	3.20	0.14–191.4	2.48 × 10^9^	1.22 × 10^6^	215.1–3.77 × 10^10^
3284^a^	2839.6	2.93	0.14–163.5	2.26 × 10^9^	6.10 × 10^5^	189.6–3.30 × 10^10^
Zh^b^	0.14	0.05	0.01–0.68	3.94	1.28	0.054–22.67
Kr^b^	0.10	0.04	0.01–0.55	1.98	0.82	0.063–10.88
Gr^b^	0.12	0.04	0.01–0.70	3.90	0.94	0.058–24.95
Ca^b^	0.12	0.04	0.01–0.68	331.8	0.85	0.064–31.76
Low Susc^c^	385.1	1.67	0.10–61.2	7.61 × 10^8^	1.24 × 10^4^	11.76–7.60 × 10^9^
High Susc^c^	0.12	0.04	0.01–0.73	21.88	0.90	0.064–50.67

*^a^Strains used in the Morgan et al. (1985) challenge study. ^b^Natural experiments or outbreaks from: Zh = Zhang et al. (2012); Kr = Krovacek et al. (1995); Gr = Granum et al. (1998); Ca = Carnahan et al. (1991). ^c^Low and high susceptibility correspond to the 1% dose calculated globally for all the strains and for the natural experiments or outbreaks, respectively.*

### Estimation of Risk

**FIGURE 6:**
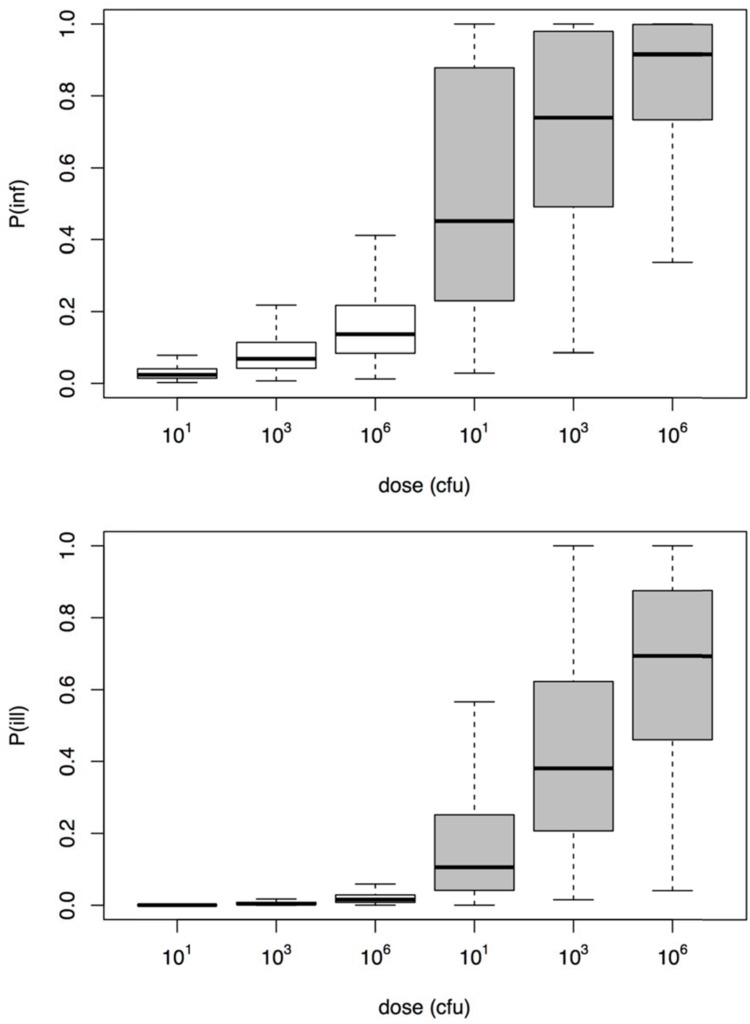
**Risk of infection (upper graphic) and illness (lower graphic) for the three different dose scenarios *Aeromonas* exposure (10, 1000, and 10^6^ cfu)**.

To illustrate the application of the dose response models derived here, a small risk study was set up, using a simple scenario of three different, fixed doses, representing low, medium, and high exposure. **Figure 6** shows the resulting risks of *Aeromonas* infection and illness. It is clear that using the challenge study dose response relation, the illness risk remains small, even for exposure to considerable doses of *Aeromonas*. On the other hand, the natural exposure events dose response relation produces high illness risks, even when the dose is moderate or low.

## Discussion

The major clinical manifestation produced by *Aeromonas* is diarrhea affecting principally young children and immunocompromised patients (Janda and Abbott, 2010; Figueras and Beaz-Hidalgo, 2015). The single available human challenge study for *Aeromonas* shows low susceptibility, in particular to developing acute enteric symptoms with only 2/57 (3.5%) challenged being positive (**Table 1**; Morgan et al., 1985). In contrast, the “natural exposure events” indicate that a high risk of illness may be associated with exposure to *Aeromonas* even at moderate to low doses. Thus, host susceptibility may be a strong determinant for the illness risk of *Aeromonas* sp. The dose response assessment presented in this study corroborates this conclusion (median 1% illness dose was ~1.4 × 10^4^ times higher in “the natural exposure events” compared to the clinical challenge), and shows how these two subsets of illness data lead to different estimates of susceptibility.

Although invaluable for understanding infection and pathogenesis, clinical challenge may not quantitatively represent natural infection that occur during an outbreak. Clinical challenge may underestimate the risk because pathogen inocula may decrease in virulence because of safety testing, often requiring repeated culture in the laboratory. Likewise, volunteers are selected for being healthy and immunocompetent to reduce the risk of serious complications. Conversely, outbreaks select for virulent pathogen strains and susceptible hosts, as this increases the probability that a cluster of cases is detected (Teunis et al., 2004). Consequently, challenge studies and outbreaks may both be biased, in opposite directions: each may represent an extreme in the continuum of dose response relations as discussed earlier (Teunis et al., 2005, 2010; Thebault et al., 2013). Outbreaks or natural exposure events may select for highest susceptibility in the affected host population producing a high illness risks, even when the dose is moderate or low. For instance in the food outbreak that produced acute diarrhea in over 200 college students’ described by Zhang et al. (2012) the estimated concentration of *Aeromonas* in the cucumber salad determined in the present study ranged from 200 to 1000 cfu/g (**Figure 1**). This estimated concentration assumed a homogeneous distribution of bacteria (Teunis et al., 2005) in the cucumber salad. As the origin of the contamination in this outbreak was attributed to rinse water of the vegetables, contaminated with sewage, occurrence of bacteria may not have been severely clustered. In case there is severe clustering, some subjects would have been exposed to high doses, while others would not have been exposed at all. Only when there is extreme clustering, the slope of the dose response relation is affected, not its location and the mean dose remains proportional to the amount of contaminated food that was ingested (Teunis et al., 2008, 2012a). As no information regarding clustering in the contaminated cucumber salad was provided by Zhang et al. (2012), any assumptions regarding clustering would be speculation.

Little is known about who is susceptible to any specific strain or pathogen in the population at large, or how many of the already considered susceptible like infants or immunocompromised would develop acute illness. It has been estimated that in developed countries the part of the population that could be susceptible to acquiring a foodborne disease would be near 20% (Lund and O’Brien, 2011; Lund, 2015). Challenge studies, on the other hand, tend to select for low susceptibility, and may represent the opposite extreme of the susceptibility spectrum. The results presented here for *Aeromonas* appear to support the same conclusion, where the outbreaks represent a worst case situation, while most challenged exposures result in low risk of infection and illness.

As indicated before the results of the Morgan et al. (1985) challenge study have been used to indicate that *Aeromonas* is not a true gastrointestinal pathogen (von Graevenitz, 2007; Janda and Abbott, 2010). However, it must be noted that 15 out of 57 (26%) were infected. The occurrence of few illness outcomes is not rare in human challenge studies. For instance in a challenge study for *Giardia lamblia* 40 subjects were challenged, with doses up to 10^6^ cysts resulting in 21 infected subjects, yet not a single of these infected subjects showed any symptoms of enteric illness (Rendtorff, 1954). Nevertheless, the significance of *Giardia* as a human enteric pathogen is not doubted. The same may be found for *Plesiomonas shigelloides* with 22 subjects challenged, resulting in eight infected and none of these becoming ill (Herrington et al., 1987). The 3.5% acute diarrhea incidence of the Morgan et al. (1985) study is similar to the 2% of adult diarrhea incidence found in several studies (Svenungsson et al., 2000; Vila et al., 2003; Figueras, 2005; Senderovich et al., 2012; Figueras and Beaz-Hidalgo, 2015).

The above analysis suggests that the natural exposures involve much lower doses than the challenge study. Notwithstanding the low risk predicted from the challenge study data, the same study suggests that the risk of infection is not extremely low, in particular for two of the strains 6Y and 3647 (Morgan et al., 1985). This means that the risk cannot be ignored, even though few cases of acute diarrhea were observed. Janda and Abbott (2010) underlined that the strain SSU (CDC diarrheal isolate), which is probably the most well-characterized *Aeromonas* strain with respect to virulence factors (i.e., carriage and expression of enterotoxin genes, and potential colonization factors see studies directed by Dr. Chopra, i.e., Grim et al., 2014 and Ponnusamy et al., 2016, and references therein) did not produce infection (colonization) nor diarrhea in the challenge study, so this cannot support the idea that the strains were wrongly selected. However, it is still possible that a critical virulence or colonization factor could have been lost when subculturing the original strains (Morgan et al., 1985; Janda and Abbott, 2010). Experimental studies in a murine animal model showed that the injection of strain SSU generated the death of all mice in 48 h (Grim et al., 2014; Ponnusamy et al., 2016). Strain SSU has been recently identified as *Aeromonas dhakensis*, a highly virulent species (Beaz-Hidalgo et al., 2013; Morinaga et al., 2013; Figueras and Beaz-Hidalgo, 2015), instead of *A. hydrophila* as originally thought (Morgan et al., 1985). It is now known that the importance attributed to *A. hydrophila* is due to misidentification of the majority of strains as belonging to this species, using phenotypic identification systems (Soler et al., 2003; Figueras, 2005; Beaz-Hidalgo et al., 2010; Morinaga et al., 2013). In fact even 30% of the genomes deposited at the GenBank database with the name of *A. hydrophila* do not belong to this species (Figueras et al., 2014; Beaz-Hidalgo et al., 2015). Old literature on clinical aeromonads limited the identification only to three species, i.e., *A. hydrophila*, *A. sobria* (the correct terminology for the clinical strains is *A. veronii* biovar sobria) and *A. caviae* (Janda and Abbott, 1998; Figueras, 2005; von Graevenitz, 2007). However, strains under these names may belong to other species (Janda and Abbott, 1998; Soler et al., 2003; Figueras, 2005). For instance in the Morgan et al. (1985) study among the five strains named *A. hydrophila* three (6Y, B158, and 3284) were suspected to belong to *A. sobria* (*A. veronii* biovar sobria), and strain SSU, as commented above, corresponds to *A. dhakensis*. A recent review showed that when using molecular identification methods ca. 96% of the recovered isolates (313/327) from human feces in different studies belong to 4 species: *A. caviae* (37.6%), *A. veronii* (27.2%), *A. dhakensis* (16.5%), and *A. hydrophila* (14.5%) (Figueras and Beaz-Hidalgo, 2015). A higher prevalence of *A. caviae* in diarrhea cases over the other species is reported also in previous reviews (von Graevenitz, 2007). However, studies on traveler’s diarrhea where an adult population is involved are dominated by *A. veronii* (Vila et al., 2003; von Graevenitz, 2007 and references therein). Also in a recent study performed by Chen et al. (2015) in and adult population from Taiwan *A. veronii* was the prevailing species (54.6%) followed by *A. caviae* (27.6%), and with one strain of each of the species *A. dhakensis* (9.1%) and *A. sanarellii* (9.1%). Interestingly one patient in the latter study had a history of eating lettuce with salad prior to the illness. In a recent study tomatoes and parsley irrigated with the same water showed the same genotype of a strain of *A. sanarellii* and the same occurred for an *A. caviae* strain recovered from the water and the irrigated lettuces (Latif-Eugenín, 2015).

As we commented in the introduction it is generally assumed that the virulence of *Aeromonas* is multifactorial and that only a subset of *Aeromonas* strains is capable of causing gastroenteritis in humans (von Graevenitz, 2007; Janda and Abbott, 2010; Beaz-Hidalgo and Figueras, 2013; Figueras and Beaz-Hidalgo, 2014; Grim et al., 2014; Ponnusamy et al., 2016). In fact some *Aeromonas* strains possess several virulence toxins and secretion systems, among which the Type III secretion system and the Shiga toxin genes are similar to those present in other important pathogenic bacteria (Figueras et al., 2007a; Alperi and Figueras, 2010; Figueras and Beaz-Hidalgo, 2014). The large variation observed among *Aeromonas* strains maybe linked to the site where the virulence is expressed (Leggett et al., 2012). For instance carriage of virulence genes that have a local action within the host cells like, i.e., the injection of toxins through the Type III or Type IV secretion systems may generate a more virulent response than other strains that exert virulence at more distant sites (Leggett et al., 2012). The latter occurs when pathogens cause secretion of proteins binding to host cells (i.e., immune modulators delivered by the general secretary pathway, or by Type I, II, and V secretory systems). This different pathway or the synergetic effect of such pathways may lead to differences in infective doses (Leggett et al., 2012). In addition the host susceptibility is very relevant, now we know that the genetic polymorphism in the host population determines the variability observed in the type or intensity of responses against the encountered specific pathogens ranging from asymptomatic infections to fatal disease (Ribet and Cossart, 2015).

The concentrations of *Aeromonas* that were selected in the exposure scenarios to assess risk of infection, were those that can normally be found in oysters, i.e., MPN 9.3/100 g or 10 cfu/100 ml have been reported in several drinking water studies (Abeyta et al., 1986; Austin et al., 1996; Figueras et al., 2005; Egorov et al., 2011; Robertson et al., 2014) or for instance in Milk (1-2 × 10^3^) or meat products (10^2^-10^3^) as found by Borrell et al. (1998). In fact a MPN 9.3/100g was the concentration of *Aeromonas* found in oysters considered to be the source of an outbreak that affected 472 persons suffering from gastroenteritis in Louisiana (Abeyta et al., 1986). Concentrations as high as 10^5^ or 10^6^ cfu/100 ml can be found in treated wastewater and in reclaimed waters used for irrigation of vegetables (Austin et al., 1996; McMahon and Wilson, 2001; Janda and Abbott, 2010; Fernandez-Cassi et al., 2016). Such sources could contaminate ready to eat vegetables and pose a risk for human health as was recently demonstrated finding the same genotype of *Aeromonas* in the irrigated water and in the irrigated vegetables as commented above (Latif-Eugenín, 2015).

The dose response assessment reported here shows that when *Aeromonas* is present, the probability of infection may not be negligible, and in a susceptible host, there may be a high risk of acute enteric illness. It is therefore important to determine how rare it is for a host to be highly susceptible. Outbreaks or other natural experiments are hard to find. This may be because they are so rare, but there could be underreporting because *Aeromonas* is often not considered among the microbes to be analyzed during investigation of outbreaks of infectious gastroenteritis (Janda and Abbott, 2010; Figueras and Beaz-Hidalgo, 2015). In fact the recognition of *Aeromonas* in the clinical setting occurs accidentally on routine enteric isolation media designed for other enteropathogens like XLD (MacConkey, Xylose Lysine Dextrose Agar), SS (Salmonella–Shigella Agar), or CIN (Cefsulodin–Irgasa–Novobiocin Agar) as described earlier (Figueras, 2005; Janda and Abbott, 1998, 2010; Figueras and Beaz-Hidalgo, 2015).

The carriage of an infectious microorganism in the general population cannot be easily extrapolated from (observable) symptomatic cases, because of underreporting, but also because in the population at large, a great majority of the infections may remain asymptomatic. Biomarker studies using serum antibodies for estimating infection (seroconversion) rates in the general population have shown that for *Campylobacter* there may be more than 1,000 asymptomatic infections for every notified case of campylobacteriosis (Teunis et al., 2012b, 2013). Similar ratios have been found for *Salmonella* (Simonsen et al., 2011). Serology could be helpful in determining whether there could be substantial carriage of *Aeromonas* in the general population. Serological evidence of infection has been provided in some cases, supporting the true enteropathogenicity of *Aeromonas* (see review by Janda and Abbott, 1998; Crivelli et al., 2001). In fact a specific secretory immunoglobulin A (sIgA) response at the intestinal mucosa against the extracellular products that appeared in the feces of patients with *Aeromonas* diarrhea was demonstrated by Crivelli et al. (2001).

Prevalence or incidence of HIV, hepatitis C have been estimate using the “evidence synthesis”, that is a well-accepted methodology for integrating various sources of data to estimate a quantity of interest for which there are no or limited direct data (McDonald et al., 2015 and references therein). This approach was applied recently to determine the incidence of symptomatic pertussis infection in the Netherlands (McDonald et al., 2015) and may help in determining whether the low numbers of symptomatic cases are consistent with frequent isolation of *Aeromonas.*

The predicted risks of infection and illness show considerable uncertainty. This is caused by the variation observed between outcomes produced by different strains, but it also results from the small sizes of the exposed groups, in particular in the challenge study (**Table 1**). Unfortunately it is not likely that there will be more or better data available, to improve the precision of the estimates. Despite the small sample sizes and uncertain doses, what is relevant is that the low and high susceptibility estimates are clearly different.

### *Aeromonas* is a True Enteropathogen

Arguments used against considering *Aeromonas* an enteropathogen, i.e., the lack of outbreaks, the non-fulfillment of the Koch’s postulates, the low numbers of acute illness in the human challenge study and the lack of animal model have all been addressed with evidence to the contrary in other studies (Chu et al., 2006; Figueras et al., 2007b; von Graevenitz, 2007; Janda and Abbott, 2010; Figueras and Beaz-Hidalgo, 2015). A murine model of *Aeromonas* diarrhea has been developed (Abuelsaad et al., 2013). The Koch’s postulates have been fulfilled by considering the incidental ingestion of *A. trota* by a laboratory worker (Carnahan et al., 1991), the challenge study of Morgan et al. (1985) and the outbreak reports included in this study (Krovacek et al., 1995; Granum et al., 1998; Zhang et al., 2012). In addition, even though few outbreaks exist, an epidemiological link has been found between the source of infection and the clinical isolates. The same *Aeromonas* strains (verified by genotyping) that caused diarrhea were isolated from drinking water (Khajanchi et al., 2010; Pablos et al., 2011), from the consumed shrimp cocktail (Altwegg et al., 1991) and from the household environment (Demarta et al., 2000). Also the same genotype of *Aeromonas* has been isolated from an HIV/AIDS patient suffering from gastroenteritis and from their household drinking-water (Ramalivhana et al., 2010).

## Concluding Remarks and Perspectives

Combined evidence collated from clinical studies in humans and outbreaks shows that *Aeromonas* should be treated as a human enteropathogen. Exposure to low doses of *Aeromonas* sp. may lead to infection, but most infections may remain asymptomatic. Given the omnipresence of *Aeromonas* in the environment, seroprevalence studies in the general population are needed to reveal frequent carriage. Further investigations are needed to determine the specific combination of host, environment and pathogen factors that lead to the occurrence of acute enteric symptoms (illness) associated with *Aeromonas* infections. The risk of illness may be considerable, even when exposed to moderate doses as shown for the studied outbreaks. Therefore, as suggested earlier, patients with underlying malignancies or immunosuppressing conditions should be closely supervised, considering the inherently high risk of invasion and dissemination associated with this population group.

## Author Contributions

PT and MF contributed equally to the conception and design of the study, PT performed the mathematical modeling and PT and MF jointly interpreted the data and wrote the manuscript. Both authors approved the final version of the paper.

## Conflict of Interest Statement

The authors declare that the research was conducted in the absence of any commercial or financial relationships that could be construed as a potential conflict of interest.

## References

[B1] Abeyta C. Kaysner C. A. Wekell M. M. Sullivan J. J. Stelma G. N. (1986). Recovery of *Aeromonas hydrophila* from oysters implicated in an outbreak of foodborne illness. *J. Food Protect.* 49 643–650. 10.4315/0362-028X-49.8.643 30959691

[B2] Abuelsaad A. S. Mohamed I. Allam G. Al-Solumani A. A. (2013). Antimicrobial and immunomodulating activities of hesperidin and ellagic acid against diarrheic *Aeromonas hydrophila* in a murine model. *Life Sci.* 93 714–722. 10.1016/j.lfs.2013.09.019 24090709

[B3] Alperi A. Figueras M. J. (2010). Human isolates of *Aeromonas* possess Shiga toxin genes (stx1 and stx2) highly similar to the most virulent gene variants of *Escherichia coli*. *Clin. Microbiol. Infect.* 16 1563–1567. 10.1111/j.1469-0691.2010.03203.x 20219084

[B4] Altwegg M. Martinetti-Lucchini G. Lüthy-Hottenstein J. Rohrbach M. (1991). *Aeromonas*-associated gastroenteritis after consumption of contaminated shrimp. *Eur. J. Clin. Microbiol. Infect. Dis.* 10 44–45. 10.1007/BF01967100 2009882

[B5] Austin B. Altwegg M. Gosling P. J. Joseph S. W. (1996). *The Genus Aeromonas.* Chichester: John Wiley & Sons, Ltd.

[B6] Beaz-Hidalgo R. Alperi A. Buján N. Romalde J. L. Figueras M. J. (2010). Comparison of phenotypical and genetic identification of *Aeromonas* strains isolated from diseased fish. *Syst. Appl. Microbiol.* 33 149–153. 10.1016/j.syapm.2010.02.002 20227844

[B7] Beaz-Hidalgo R. Figueras M. J. (2013). *Aeromonas* spp. whole genomes and virulence factors implicated in fish disease. *J. Fish Dis.* 36 371–388. 10.1111/jfd.12025 23305319

[B8] Beaz-Hidalgo R. Hossain M. J. Liles M. R. Figueras M. J. (2015). Strategies to avoid wrongly labelled genomes using as example the detected wrong taxonomic affiliation for *Aeromonas* genomes in the GenBank database. *PLoS ONE* 10:e0115813 10.1371/journal.pone.0115813 PMC4301921 25607802

[B9] Beaz-Hidalgo R. Martinez-Murcia A. J. Figueras M. J. (2013). Reclassification of *Aeromonas hydrophila* subsp. *dhakensis* Huys et al. 2002 and *Aeromonas aquariorum* Martinez-Murcia et al. 2008 as *Aeromonas dhakensis* sp. nov. comb nov. and emendation of the species *Aeromonas hydrophila*. *Syst. Appl. Microbiol.* 36 171–176. 10.1016/j.syapm.2012.12.007 23485124

[B10] Borrell N. Figueras M. J. Guarro J. (1998). Phenotypic identification of *Aeromonas* genomospecies from clinical and environmental sources. *Can. J. Microbiol.* 44 103–118. 10.1139/w97-135 9575026

[B11] Carnahan A. M. Chakraborty T. Fanning G. R. Verma D. Ali A. Janda J. M. (1991). *Aeromonas* trota sp. nov., an ampicillin-susceptible species isolated from clinical specimens. *J. Clin. Microbiol.* 29 1206–1210. 1864939 10.1128/jcm.29.6.1206-1210.1991 PMC269970

[B12] Chen P. L. Tsai P. J. Chen C. S. Lu Y. C. Chen H. M. Lee N. Y. (2015). *Aeromonas* stool isolates from individuals with or without diarrhea in southern Taiwan: predominance of *Aeromonas veronii*. *J. Microbiol. Immunol. Infect.* 48 618–624. 10.1016/j.jmii.2014.08.007 25440979

[B13] Chu Y. W. Wong C. H. Tsang G. K. L. Kwok M. S. W. Wong R. K. O. Lo J. Y. C. (2006). Lack of association between presentation of diarrhoeal symptoms and fecal isolation of *Aeromonas* spp. amongst outpatients in Hong Kong. *J. Med. Microbiol.* 55 349–351. 10.1099/jmm.0.46266-0 16476802

[B14] Crivelli C. Demarta A. Peduzzi R. (2001). Intestinal secretory immunoglobulin A (sIgA) response to *Aeromonas* exoproteins in patients with naturally acquired *Aeromonas* diarrhea. *FEMS Immunol. Med. Microbiol.* 30 31–35. 10.1111/j.1574-695X.2001.tb01546.x 11172988

[B15] Demarta A. Tonolla M. Caminada A. Beretta M. Peduzzi R. (2000). Epidemiological relationships between *Aeromonas* strains isolated from symptomatic children and household environments as determined by ribotyping. *Eur. J. Epidemiol.* 16 447–453. 10.1023/A:1007675424848 10997832

[B16] Denny J. E. Powell W. L. Schmidt N. W. (2016). Local and long-distance calling: conversations between the gut microbiota and intra- and extra-gastrointestinal tract infections. *Front. Cell. Infect. Microbiol.* 6:41 10.3389/fcimb.2016.00041 PMC4826874 27148490

[B17] DuPont H. L. Hornick R. B. Dawkins A. T. Snyder M. J. Formal S. B. (1969). The response of man to virulent *Shigella flexneri* 2a. *J. Infect. Dis.* 119 296–299. 10.1093/infdis/119.3.296 5780532

[B18] Egorov A. I. Best J. M. Frebis C. P. Karapondo M. S. (2011). Occurrence of *Aeromonas* spp. in a random sample of drinking water distribution systems in the USA. *J. Water Health.* 9 785–798. 10.2166/wh.2011.169 22048437

[B19] Fernandez-Cassi X. Silvera C. Cervero-Aragó S. Rusiñol M. Latif-Eugeni F. Bruguera-Casamada C. (2016). Evaluation of the microbiological quality of reclaimed water produced from a lagooning system. *Environ. Sci. Pollut. Res. Int.* 10.1007/s11356-016-6812-0 [Epub ahead of print]. 27194016

[B20] Figueras M. J. (2005). Clinical relevance of *Aeromonas*. *Rev. Med. Microbiol.* 16 145–153. 10.1097/01.revmedmi.0000184410.98677.8a

[B21] Figueras M. J. Aldea M. J. Fernández N. Aspíroz C. Alperi A. Guarro J. (2007a). *Aeromonas* hemolytic uremic syndrome. A case and a review of the literature. *Diagn. Microbiol. Infect. Dis.* 58 231–234. 10.1016/j.diagmicrobio.2006.11.023 17300902

[B22] Figueras M. J. Beaz-Hidalgo R. (2014). “*Aeromonas,” in Encyclopedia of Food Microbiology* eds Batt C. Tortorello M. L. (Oxford: Elsevier Ltd) 25–30.

[B23] Figueras M. J. Beaz-Hidalgo R. (2015). “*Aeromonas* infections in humans,” in *Aeromonas* ed. Graf J. (Norfolk: Caister Academic Press) 65–108.

[B24] Figueras M. J. Beaz-Hidalgo R. Hossain M. J. Liles M. R. (2014). Taxonomic affiliation of new genomes should be verified using average nucleotide identity and multilocus phylogenetic analysis. *Genome Announc.* 2: e927-14 10.1128/genomeA.00927-14 PMC4256179 25477398

[B25] Figueras M. J. Borrego J. J. (2010). New perspectives in monitoring drinking water microbial quality. *Int. J. Environ. Res. Public Health* 7 4179–4202. 10.3390/ijerph7124179 21318002 PMC3037048

[B26] Figueras M. J. Horneman A. J. Martinez-Murcia A. Guarro J. (2007b). Controversial data on the association of *Aeromonas* with diarrhoea in a recent Hong Kong study. *J. Med. Microbiol.* 56 996–998. 10.1099/jmm.0.47062-0 17577068

[B27] Figueras M. J. Suarez-Franquet A. Chacón M. R. Soler L. Navarro M. Alejandre C. (2005). First record of the rare species *Aeromonas culicicola* from a drinking water supply. *Appl. Environ. Microbiol.* 71 538–541. 10.1128/AEM.71.1.538-541.2005 15640231 PMC544204

[B28] Graf J. (2015). *Aeromonas.* Norfolk: Caister Academic Press.

[B29] Granum P. E. O’Sullivan K. Tomas J. M. Ormen O. (1998). Possible virulence factors of *Aeromonas* spp. from food and water. *FEMS Immunol. Med. Microbiol.* 21 131–137. 10.1111/j.1574-695X.1998.tb01158.x 9685002

[B30] Grim C. J. Kozlova E. V. Ponnusamy D. Fitts E. C. Sha J. Kirtley M. L. (2014). Functional genomic characterization of virulence factors from necrotizing fasciitis-causing strains of *Aeromonas hydrophila*. *Appl. Environ. Microbiol.* 80 4162–4183. 10.1128/AEM.00486-14 24795370 PMC4068668

[B31] Herrington D. Tzipori S. Robins-Browne R. Tall B. Levine M. (1987). In vitro and in vivo pathogenicity of *Plesiomonas shigelloides*. *Infect. Immun.* 55 979–985. 3557621 10.1128/iai.55.4.979-985.1987 PMC260448

[B32] Holmberg S. D. Schell W. Fanning G. Wachsmuth I. Hickman-Brenner F. Blake P. (1986). *Aeromonas* intestinal infections in the United States. *Ann. Int. Med.* 105 683–689. 10.7326/0003-4819-105-5-690 3767148

[B33] Janda J. M. Abbott S. L. (1998). Evolving concepts regarding the genus *Aeromonas*: an expanding panorama of species, disease presentation, and unanswered questions. *Clin. Infect. Dis.* 27 332–344. 10.1086/514652 9709884

[B34] Janda J. M. Abbott S. L. (2010). The genus *Aeromonas*: taxonomy, pathogenicity, and infection. *Clin. Microbiol. Rev.* 23 35–73. 10.1128/CMR.00039-09 20065325 PMC2806660

[B35] Katz M. J. Parrish N. M. Belani A. Shah M. (2015). Recurrent *Aeromonas* bacteremia due to contaminated well water. *Open Forum Infect. Dis.* 2:ofv142 10.1093/ofid/ofv142 PMC4612566 26495324

[B36] Khajanchi B. K. Fadl A. A. Borchardt M. A. Berg R. L. Horneman A. J. Stemper M. E. (2010). Distribution of virulence factors and molecular fingerprinting of *Aeromonas* species isolates from water and clinical samples: suggestive evidence of water-to-human transmission. *Appl. Environ. Microbiol.* 76 2313–2325. 10.1128/AEM.02535-09 20154106 PMC2849238

[B37] Krovacek K. Dumontet S. Eriksson E. Baloda S. B. (1995). Isolation, and virulence profiles, of *Aeromonas hydrophila* implicated in an outbreak of food poisoning in Sweden. *Microbiol. Immunol.* 39 655–661. 10.1111/j.1348-0421.1995.tb03253.x 8577278

[B38] Latif-Eugenín F. (2015). *Aeromonas, Un Microorganismo Ambiental de Importancia en Salud Humana y Animal.* Ph.D. thesis, Universitat Rovira I Virgili, Tarrogona Available at: http://www.tdx.cat/bitstream/handle/10803/334686/Tesi%20Fadua.pdf?sequence=1&isAllowed=y

[B39] Leggett H. C. Cornwallis C. K. West S. A. (2012). Mechanisms of pathogenesis, infective dose and virulence in human parasites. *PLoS Pathog.* 8:e1002512 10.1371/journal.ppat.1002512 PMC3280976 22359500

[B40] Lund B. M. (2015). Microbiological food safety for vulnerable people. *Int. J. Environ. Res. Public Health* 12 10117–10132. 10.3390/ijerph120810117 26308030 PMC4555333

[B41] Lund B. M. O’Brien S. J. (2011). The occurrence and prevention of foodborne disease in vulnerable people. *Foodborne Pathog. Dis.* 8 961–973. 10.1089/fpd.2011.0860 21561383 PMC3159107

[B42] Martin Delgado M. Matute Cruz P. Nuñez Gallo D. Fernández González C. López González Coviella N. Valladares Hernández B. (2001). Diarrea del viajero asociada a *Aeromonas hydrophila*. *Rev. Salud Ambient.* 1 30–35.

[B43] McDonald S. A. Teunis P. van der Maas N. de Greef S. de Melker H. Kretzschmar M. (2015). An evidence synthesis approach to estimating the incidence of symptomatic pertussis infection in the Netherlands, 2005-2011. *BMC Infect. Dis.* 15:588 10.1186/s12879-015-1324-y PMC4696101 26715486

[B44] McMahon M. A. Wilson I. G. (2001). The occurrence of enteric pathogens and *Aeromonas* species in organic vegetables. *Int. J. Food Microbiol.* 70 155–162. 10.1016/S0168-1605(01)00535-9 11759753

[B45] Morgan D. R. Johnson P. C. DuPont H. L. Satterwhite T. K. Wood L. V. (1985). Lack of correlation between known virulence properties of *Aeromonas hydrophila* and enteropathogenicity for humans. *Infect. Immun.* 50 62–65. 4044042 10.1128/iai.50.1.62-65.1985 PMC262135

[B46] Morinaga Y. Yanagihara K. Latif Eugenin F. L. Beaz-Hidalgo R. Figueras M. J. (2013). Identification errors of *Aeromonas* species: the considerable virulence of the new species *Aeromonas aquariorum*. *Diag. Microbiol. Infect. Dis.* 76 106–109. 10.1016/j.diagmicrobio.2013.01.019 23461831

[B47] Mosser T. Talagrand-Reboul E. Colston S. M. Graf J. Figueras M. J. Jumas-Bilak E. (2015). Exposure to pairs of *Aeromonas* strains enhances virulence in the *Caenorhabditis elegans* infection model. *Front. Microbiol.* 6:1218 10.3389/fmicb.2015.01218 PMC4631986 26583012

[B48] Pablos M. Huys G. Cnockaert M. Rodríguez-Calleja J. M. Otero A. Santos J. A. (2011). Identification and epidemiological relationships of *Aeromonas* isolates from patients with diarrhea, drinking water and foods. *Int. J. Food Microbiol.* 147 203–210. 10.1016/j.ijfoodmicro.2011.04.006 21550680

[B49] Ponnusamy D. Kozlova E. V. Sha J. Erova T. E. Azar S. R. Fitts E. C. (2016). Cross-talk among flesh-eating *Aeromonas hydrophila* strains in mixed infection leading to necrotizing fasciitis. *Proc. Natl. Acad. Sci. U.S.A.* 113 722–727. 10.1073/pnas.1523817113 26733683 PMC4725515

[B50] Ramalivhana J. N. Obi C. L. Samie A. Labuschagne C. Weldhagen G. F. (2010). Random amplified polymorphic DNA typing of clinical and environmental *Aeromonas hydrophila* strains from Limpopo province, South Africa. *J. Health Popul. Nutr.* 28 1–6. 20214080 10.3329/jhpn.v28i1.4517 PMC2975840

[B51] Rendtorff R. C. (1954). The experimental transmission of human intestinal protozoan parasites I. *Giardia lamblia* cysts given in capsules. *Am. J. Hyg.* 59 209–220. 13138586 10.1093/oxfordjournals.aje.a119634

[B52] Ribet D. Cossart P. (2015). How bacterial pathogens colonize their hosts and invade deeper tissues. *Microbes Infect.* 17 173–183. 10.1016/j.micinf.2015.01.004 25637951

[B53] Robertson B. K. Harden C. Selvaraju S. B. Pradhan S. Yadav J. S. (2014). Molecular detection, quantification and toxigenicity profiling of *Aeromonas* spp. in source and drinking water. *Open Microbiol. J.* 8 32–39. 10.2174/1874285801408010032 24949108 PMC4062929

[B54] Senderovich Y. Ken-Dror S. Vainblat I. Blau D. Izhaki I. Halpern M. (2012). A molecular study on the prevalence and virulence potential of *Aeromonas* spp. recovered from patients suffering from diarrhea in Israel. *PLoS ONE* 7:e30070 10.1371/journal.pone.0030070 PMC3280246 22355306

[B55] Simonsen J. Teunis P. van Pelt W. van Duynhoven Y. Krogfelt K. Sadkowska-Todys M. (2011). Usefulness of seroconversion rates for comparing infection pressures between countries. *Epidemiol. Infect.* 139 636–643. 10.1017/S0950268810000750 20380770

[B56] Soler L. Marco F. Vila J. Chacón M. R. Guarro J. Figueras M. J. (2003). Evaluation of two miniaturized systems, MicroScan W/A and BBL Crystal E/NF, for identification of clinical isolates of *Aeromonas* spp. *J. Clin. Microbiol.* 41 5732–5734. 10.1128/JCM.41.12.5732-5734.2003 14662969 PMC309027

[B57] Svenungsson B. Lagergren A. Ekwall E. Evengård B. Hedlund K. O. Kärnell A. (2000). Enteropathogens in adult patients with diarrhea and healthy control subjects: a 1-year prospective study in a Swedish clinic for infectious diseases. *Clin. Infect. Dis.* 30 770–778. 10.1086/313770 10816147

[B58] Teunis P. Falkenhorst G. Ang W. Strid M. de Valk H. Sadkowska-Todys M. (2013). *Campylobacter* seroconversion rates in selected countries in the European Union. *Epidemiol. Infect.* 141 2051–2057. 10.1017/S0950268812002774 23228443 PMC9151417

[B59] Teunis P. Kasuga F. Fazil A. Ogden I. Rotariu O. Strachan N. (2010). Dose response modeling of *Salmonella* using outbreak data. *Int. J. Food Microbiol.* 144 243–249. 10.1016/j.ijfoodmicro.2010.09.026 21036411

[B60] Teunis P. Ogden I. Strachan N. (2008). Hierarchical dose response of *E. coli* O157:H7 from human outbreaks incorporating heterogeneity in exposure. *Epidemiol. Infect.* 136 761–770. 10.1017/S0950268807008771 17672927 PMC2870861

[B61] Teunis P. Takumi K. Shinagawa K. (2004). Dose response for infection by *Escherichia coli* O157:H7 from outbreak data. *Risk Anal.* 24 401–407. 10.1111/j.0272-4332.2004.00441.x 15078310

[B62] Teunis P. van den Brandhof W. Nauta M. Wagenaar J. van den Kerkhof H. van Pelt W. (2005). A reconsideration of the *Campylobacter* dose-response relation. *Epidemiol. Infect.* 133 583–592. 16050502 10.1017/s0950268805003912 PMC2870284

[B63] Teunis P. van der Heijden O. G. van der Giessen J. W. B. Havelaar A. H. (1996). *The Dose-Response Relation in Human Volunteers for Gastro–Intestinal Pathogens.* Report nr 284550002 89 Bilthoven: Rijksinstituut voor Volksgezondheid en Milieu.

[B64] Teunis P. F. Koningstein M. Takumi K. van der Giessen J. W. (2012a). Human beings are highly susceptible to low doses of *Trichinella* spp. *Epidemiol. Infect.* 140 210–218. 10.1017/S0950268811000380 21489335

[B65] Teunis P. F. van Eijkeren J. Ang W. van Duynhoven Y. Simonsen J. Strid M. (2012b). Biomarker dynamics: estimating infection rates from serological data. *Stat. Med.* 31 2240–2248. 10.1002/sim.5322 22419564

[B66] Teunis P. F. M. Havelaar A. H. (2000). The Beta Poisson model is not a single hit model. *Risk Anal.* 20 511–518. 10.1111/0272-4332.204048 11051074

[B67] Teunis P. F. M. Nagelkerke N. J. D. Haas C. N. (1999). Dose response models for infectious gastroenteritis. *Risk Anal.* 19 1251–1260. 10.1023/A:1007055316559 10765461

[B68] Thebault A. Teunis P. Le Pendu J. Le Guyader S. Denis J.-B. (2013). Infectivity of GI and GII noroviruses established from oyster related outbreaks. *Epidemics* 5 98–110. 10.1016/j.epidem.2012.12.004 23746803

[B69] Ventura R. J. Muhi E. de los Reyes V. C. Sucaldito M. N. Tayag E. (2015). A community-based gastroenteritis outbreak after Typhoon Haiyan, Leyte, Philippines, 2013. *Western Pac. Surveill. Response J.* 6 1–6. 10.2471/WPSAR.2014.5.1.010 25960917 PMC4410107

[B70] Vila J. Ruiz J. Gallardo F. Vargas M. Soler L. Figueras M. J. (2003). Aeromonas spp. and traveler’s diarrhea: clinical features and antimicrobial resistance. *Emerg. Infect. Dis.* 9 552–555. 10.3201/eid0905.020451 12737738 PMC2972757

[B71] von Graevenitz A. (2007). The role of *Aeromonas* in diarrhea: a review. *Infection* 35 59–64. 10.1007/s15010-007-6243-4 17401708

[B72] Wadhwa S. G. Khaled G. H. Edberg S. C. (2012). Comparative microbial character of consumed food and drinking water. *Crit. Rev. Microbiol.* 28 249–279. 10.1080/1040-840291046740 12385500

[B73] Zhang Q. Shi G. Q. Tang G. P. Zou Z. T. Yao G. H. Zeng G. (2012). A foodborne outbreak of *Aeromonas hydrophila* in a college, Xingyi City, Guizhou, China, 2012. *Western Pac. Surveill. Response J.* 3 39–43. 10.5365/WPSAR.2012.3.4.018 23908938 PMC3729099

