# Supplementary material for: Reassessment of the Enteropathogenicity of Mesophilic Aeromonas Species
Source: Front Microbiol. 2016 Sep 21;7:1395. doi: 10.3389/fmicb.2016.01395 (PMC5030306; doi:10.3389/fmicb.2016.01395)
Supplement: Supplementary file 1 [file Data_Sheet_1.PDF]

Supplementary Material

Reassessment of the enteropathogenicity of *Aeromonas*

Peter Teunis and Maria José Figueras*

*** Correspondence: mariajose.figueras@urv.cat**

1 Supplementary Data

Dose response assessment

There are two stages in the data analysis: any exposed (challenged) subject may become infected with a probability P_{inf} and any infected subject may develop acute enteric symptoms with a probability $P_{\text{ill|inf}}$. Both of these probabilities depend on the dose. The dose response relation for infection

$$P_{inf}(cV) = {}_1F_1(\alpha, \alpha+\beta, -cV)$$

[Teunis et al. 2000]. Parameters are transformed as below

$$u_1 = \frac{\alpha}{\alpha+\beta} \quad w_1 = \log\left(\frac{u_1}{1-u_1}\right)$$
$$v_1 = \alpha+\beta \quad z_1 = \log(v_1)$$

so that w_1 is a measure of infectivity (location) and z_1 a measure of variation in infectivity (spread). The conditional probability of illness among infected subjects $P_{\text{ill|inf}}$ is modeled as

$$P_{ill \vee inf} = 1 - \left(1 + \frac{cV}{\eta}\right)^{-r}$$

the hazard model of illness dose response [Teunis et al., 1999]. Parameter transformation identical to the infection parameters

$$u_2 = \frac{r}{r+\eta} \quad w_2 = \log\left(\frac{u_2}{1-u_2}\right)$$
$$v_2 = r+\eta \quad z_2 = \log(v_2)$$

again translates the parameters r and η into a location parameter w_2 and a spread parameter z_2.

Parameters were estimated by pathogen strain or outbreak. Parameter estimates (posterior means and 95% credible intervals) are listed in Table 1. The parameters for location of infectivity and pathogenicity (w_1 and w_2) were given normal priors with means μ_{w1}, μ_{w2} and fixed precision τ_w. μ_{w1} and μ_{w2} were assumed to be normally distributed, with separate distributions for outbreaks and for

different strains in the challenge study, to account for variation, among outbreaks and challenge study strains, respectively. The parameters for variation (spread) in infectivity z_1, and in pathogenicity z_2, were given normal priors with fixed means and precision, not differentiating between strain and host effects.

Uncertainty in applied doses was incorporated by using informative priors, assuming a 95% interval for the dose measured 1 unit on a $_{10}$log scale, both for the challenge study and the outbreaks, except for the outbreak reported by Zhang et al. 2012, for which the concentration of the pathogens in the salad was unknown. This was accounted for by taking up the concentration as an additional parameter, to be estimated jointly with the dose response parameters (Teunis et al. 2005) and using a flat prior.

The model was specified and run in JAGS (v4.2.0) (Plummer, 2003) from R (v3.2.4) [R core team, 2015] using rjags (v4-6) [Plummer, 2013]. Three Markov chains were run with each 10^5 iterations, with thinning to produce a posterior sample of size 3,000 for each parameter (and the log-concentrations). Convergence was checked with Gelman-Rubin diagnostic, for the transformed parameters (w and z), and the (log) concentrations. Hyperpriors for the location parameters w_1 and w_2 were N(0,0.1) (mean, precision), both for the challenge studies ("low virulence") and the outbreak studies ("high virulence"). Priors for the spread parameters z_1 and z_2 were N(0,0.5). Hyperpriors for the variation (precision) of $w_{1,2}$ and $z_{1,2}$ among individual studies were Gamma(5,5). JAGS source code is available on request from PT.

References

Plummer M. JAGS: A program for analysis of Bayesian graphical models using Gibbs sampling. In: Proceedings of the 3rd International Workshop on Distributed Statistical Computing (DSC 2003), Vienna, Austria. 2003; 1–10.

Plummer M. rjags: Bayesian graphical models using MCMC, 2013. R package.

R Core Team. R: A Language and Environment for Statistical Computing. R Foundation for Statistical Computing, Vienna, Austria, 2015.

2 Supplementary Table

TABLE 1. Estimated dose response parameters, for all strains/natural experiments separately, and for the low/high susceptibility subsets.

	α		β		r		$1/\eta$	
	Mean	95% CI	Mean	95% CI	Mean	95% CI	Mean	95% CI
6Y[a]	0.032	0013 – 0.061	3.008	0.126 – 15.60	0.258	0.001 – 0.086	6.039	0.143 – 32.62
B158[a]	0.008	0.002 – 0.021	1.468	0.070 – 7.681	0.252	0.001 – 0.161	6.355	0.142 – 34.51
3647[a]	0.013	0.004 – 0.028	1.889	0.082 – 9.882	0.248	0.001 – 0.153	6.691	0.158 – 36.46
SSU[a]	0.007	0.002 – 0.018	1.368	0.065 – 6.963	0.248	0.001 – 0.159	6.239	0.143 – 35.33
3248[a]	0.008	0.002 – 0.020	1.406	0.066 – 7.428	0.262	0.001 – 0.154	6.269	0.146 – 35.19
Zh	0.502	0.030 – 3.647	1.193	0.001 – 7.234	1.675	0.048 – 13.50	4.640	0.009 – 27.86
Kr[b]	0.535	0.056 – 3.604	1.390	0.001 – 8.267	1.715	0.078 – 13.40	5.314	0.010 – 31.72
Gr[b]	0.521	0.031 – 3.623	1.254	0.001 – 7.655	1.700	0.041 – 13.46	4.977	0.009 – 29.55
Ca[b]	0.532	0.028 – 3.640	1.329	0.001 – 7.927	1.730	0.037 – 13.42	5.224	0.009 – 30.60
Low Susc[c]	0.013	0.002 – 0.043	1.811	0.078 – 9.683	0.264	0.001 – 0.171	6.598	0.145 – 36.38
High Susc[c]	0.522	0.024 – 4.534	1.301	0.001 – 7.976	1.700	0.030 – 13.34	5.065	0.009 – 30.33

[a]Strains used in the Morgan et al. (1985) challenge study.

[b]Natural experiments or outbreaks from: Zh=Zhang et al. (2012); Kr=Krovacek et al. (1995); Gr=Granum et al. (1998); Ca=Carnahan et al. (1991).

[c]Low and high susceptibility correspond to the global dose response calculated for all the strains together and for the natural experiments or outbreaks, respectively.
